# Long non-coding RNAs function as novel predictors and targets of non-small cell lung cancer: a systematic review and meta-analysis

**DOI:** 10.18632/oncotarget.23994

**Published:** 2018-01-04

**Authors:** Yanlu Xiong, Tao Wang, Mingxing Wang, Jinbo Zhao, Xiaofei Li, Zhipei Zhang, Yongsheng Zhou, Jiabao Liu, Lintao Jia, Yong Han

**Affiliations:** ^1^ Department of Thoracic Surgery, Tangdu Hospital, Fourth Military Medical University, Xi'an, China; ^2^ State Key Laboratory of Cancer Biology, Department of Biochemistry and Molecular Biology, Fourth Military Medical University, Xi'an, China; ^3^ Department of Neurobiology and Collaborative Innovation Center for Brain Science, School of Basic Medicine, Fourth Military Medical University, Xi'an, China

**Keywords:** lncRNA, NSCLC, biomarker, prognosis, malignancy

## Abstract

**Objectives:**

Non-small cell lung cancer (NSCLC) is associated with high morbidity and mortality, leading the understanding the pathogenesis paramount. Recent studies suggest that long non-coding RNAs (lncRNAs) play an important role in NSCLC. We conducted a systematic review to examine the relationship between lncRNAs and NSCLC.

**Materials and Methods:**

We calculated hazard ratios (HRs) and 95% confidence intervals (CIs) to estimate overall survival (OS), and odds ratios (ORs) and 95% CIs to assess clinicopathological parameters. Also, pooled sensitivity and specificity values were used to measure the diagnostic value of lncRNAs for NSCLC. Finally, we summarized the molecular mechanisms underlying the activity of lncRNAs in NSCLC.

**Results:**

We found that high expression of oncogenic lncRNAs was associated with a poor prognosis (OS: HR, 1.18; 95% CI, 1.14–1.22) and poor clinicopathological characteristics (tumor size: OR, 2.74 or 2.04; 95% CI, 1.66–4.52 or 1.09–3.79 based on the two classification criterias; lymph node metastasis: OR, 3.30; 95% Cl, 2.42–4.49), Also, high expression of tumor-suppressor lncRNAs was correlated with longer survival times (OS: HR, 0.54; 95% CI, 0.44–0.66) and improved clinical characteristics (tumor size: OR, 0.33 or 0.28; 95% CI, 0.14–0.75 or 0.18–0.45; lymph node metastasis: OR, 0.37; 95% Cl, 0.26–0.52). Furthermore, we found that lncRNAs could be used as serum biomarkers of NSCLC (sensitivity: 0.81; 95% CI, 0.72–0.87; specificity: 0.83; 95% CI, 0.73–0.90). Finally, lncRNAs regulated expression of key proteins, thereby mediating development of a malignant phenotype.

**Conclusions:**

lncRNAs have significant clinical value in NSCLC and could function as novel predictors of disease and/or as therapeutic targets.

## INTRODUCTION

Lung cancer is a leading cause of cancer-related death worldwide, with a 5 year survival rate of about 15% [1]. Non-small cell lung cancer (NSCLC) accounts for approximately 80% of lung cancers, whose ravages has intensely hijacked welfare of human beings for the tremendous burden on health and economy [2, 3]. However, the complexity of the pathogenic mechanisms involved means that effective therapies are difficult to design; therefore, a deeper understanding of the molecular pathways underlying such malignancy is essential [4, 5].

Cancer possesses several malignant hallmarks caused by disorganized cellular signaling networks, owing to the dysfunction of critical genes in cellular processes [6]. Recently, attributing to unique physiochemical properties like flexibility in recognizing ability and prevalence in distributing, long non-coding RNAs (lncRNAs) have emerged to have pivotal function in cancer biology mainly via regulating oncogenes or tumor suppressor genes [7–10]. Moreover, a myriad of studies highlighted the relationship between lncRNAs and NSCLC, and suggested that lncRNAs could play a momentous role in modulating malignant progression, presented to be a novel breakpoint to handle NSCLC [11, 12].

However, a single study involved a single lncRNA was not a strong platform on which to base an evaluation of the clinical value of lncRNAs in NSCLC. Therefore, we undertook a systematic review of the relationship between lncRNAs and the clinicopathological characteristics of NSCLC. We found that expression of lncRNA was associated with prognosis, tumor size, and lymph node metastasis. Furthermore, lncRNAs in serum may have utility as a diagnostic biomarker for NSCLC. Last, we summarized the molecular mechanisms about these lncRNAs in NSCLC. Taken together, these findings highlighted the clinical value of lncRNAs in NSCLC and promoted research into how lncRNAs modulate carcinogenesis of NSCLC.

## RESULTS

### Data extraction

**Figure 1:**
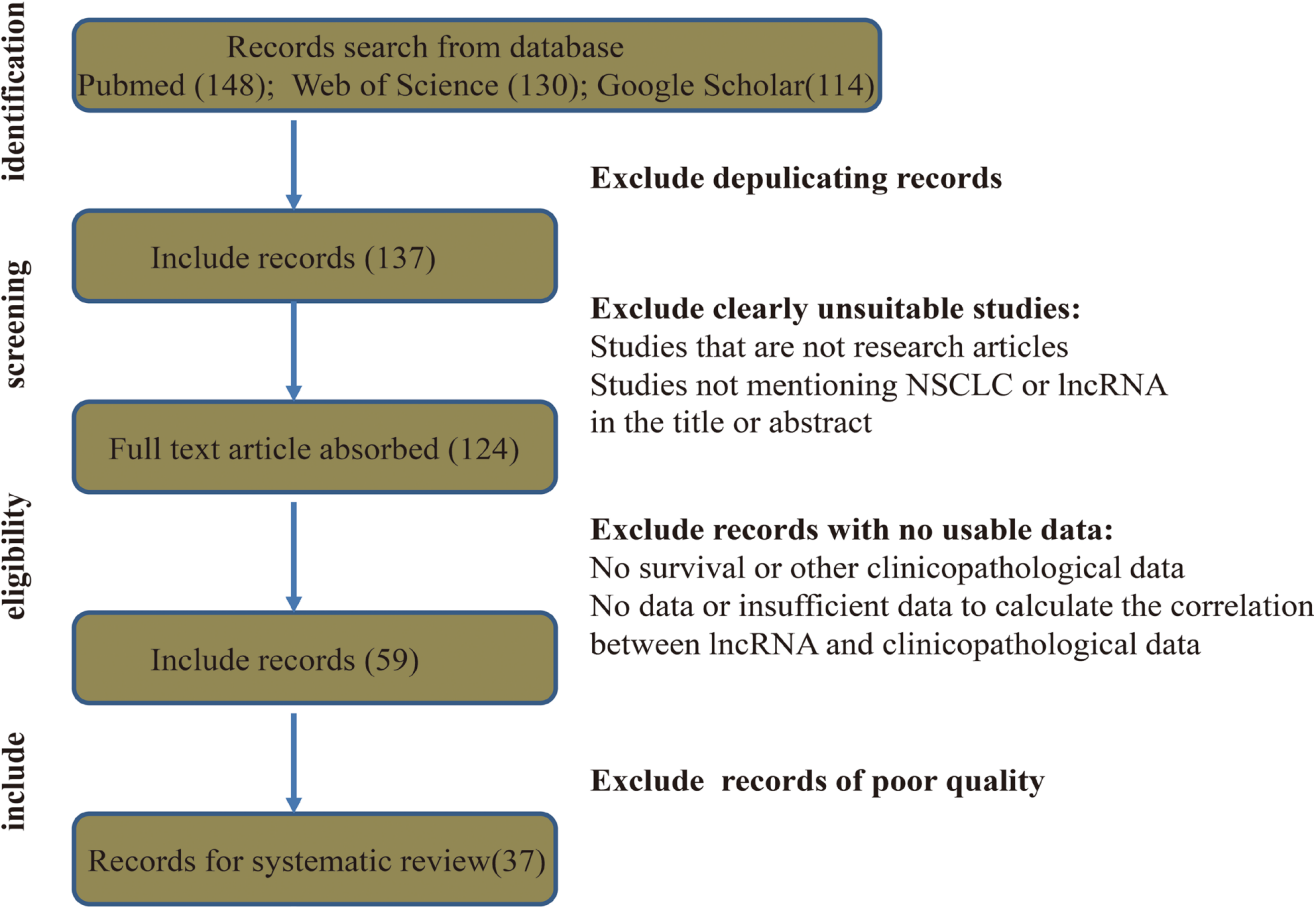
Flow diagram showing the study collection process

Up to 392 publications were identified. After excluding duplicates, the titles and abstracts of 137 studies were reviewed and irrelevant studies were excluded. Full text versions of the remaining 124 studies were examined, and those with insufficient data were excluded. Next, 59 studies were subjected to quality assessment. Finally, 37 studies were subjected to systematic review (Figure 1).

### LncRNA expression is associated with NSCLC prognosis

**Figure 2:**
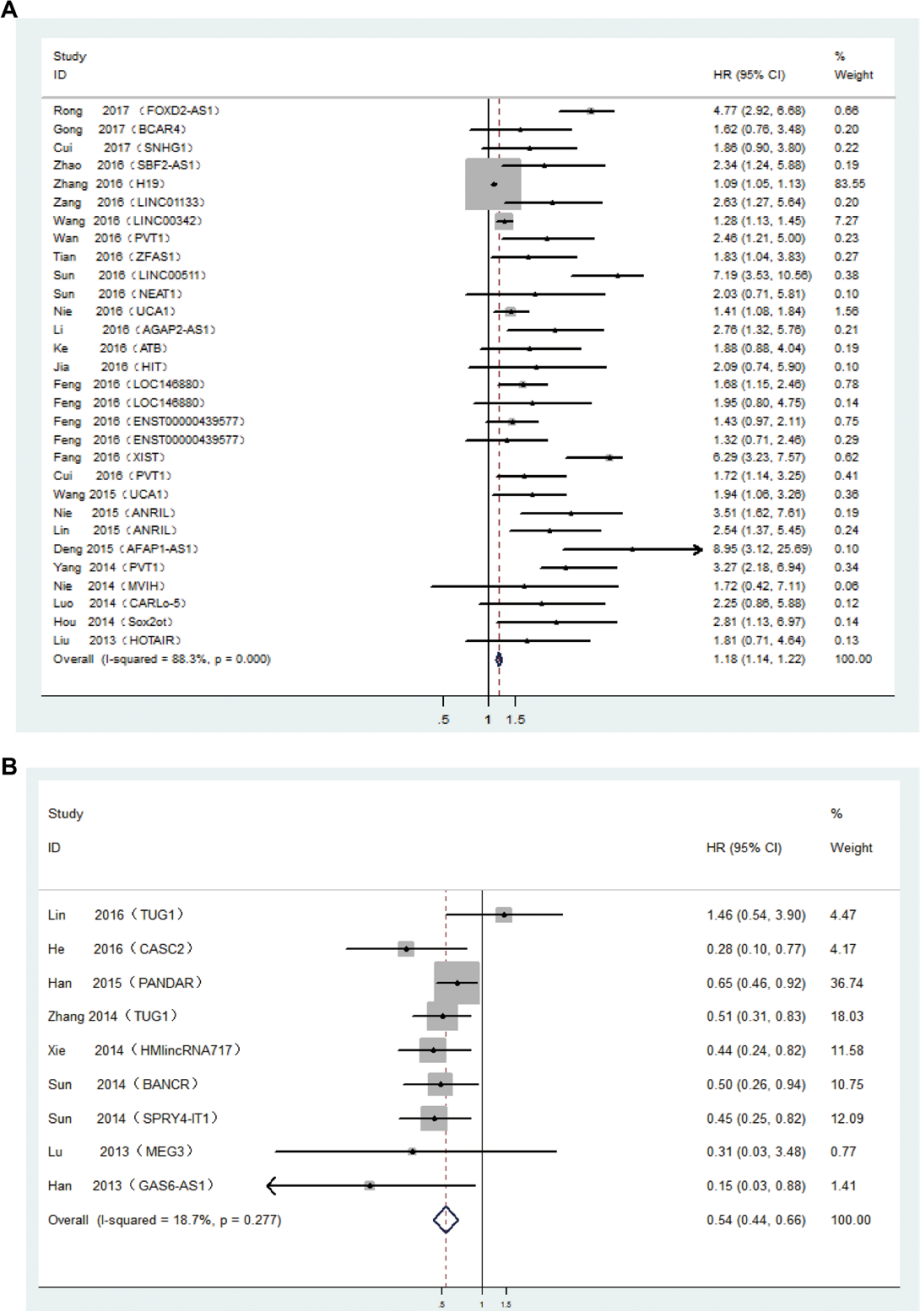
(**A**) Forest plot showing the hazard ratios and 95% confidence intervals for OS according to the type of oncogenic lncRNAs. (**B**) Forest plot showing the hazard ratios and 95% confidence intervals for OS according to the type of tumour suppressor lncRNAs.

First, we constructed a Table, including author name, publication year, cohort and country, lncRNA type, data type, sample size (exhibiting whole numbers of patients and numbers of groups with high or low expression based on the original classification), Expression file (relative expression of tumor tissues compared to corresponding normal tissues), hazard ratios (HRs), and 95% confidence intervals (CIs) (Supplementary Table 1). After carefully reviewing the medical records with respect to lncRNA and NSCLC, lncRNAs were divided into two groups: oncogenic lncRNAs and tumour suppressor lncRNAs. Next, a separate meta-analysis was performed to estimate the relationship between these two types of lncRNAs and overall survival (OS) of patients diagnosed with NSCLC. We found that 24 lncRNAs (FOXD2-AS1 [13], BCAR4 [14], SNHG1 [15], SBF2-AS1 [16], H19 [17], LINC01133 [18], LINC00342 [19], PVT1 [20–22], ZFAS1 [23], LINC00511 [24], NEAT1 [25], UCA1 [26, 27], AGAP2-AS1 [28], ATB [29], HIT [30], LOC146880 [31], ENST00000439577 [31], XIST [32], ANRIL [33, 34], AFAP1-AS1 [35], MVIH [36], CARLo-5 [37], Sox2ot [38], HOTAIR [39]) were oncogenic (HR, 1.18; 95% Cl, 1.14–1.22; Figure 2A), whereas eight (TUG1 [40, 41], CASC2 [42], PANDAR [43], HMlincRNA717 [44], BANCR [45], SPRY4-IT1 [46], MEG3 [47], GAS6-AS1 [48]) were tumour-suppressor lncRNAs(HR, 0.54; 95% Cl, 0.44–0.66; Figure 2B).

### The type of lncRNA affects the malignant clinicopathologic characteristics of NSCLC

We performed another meta-analysis to further investigate the correlation between lncRNA expression and malignant clinicopathologic characteristics of NSCLC. We first summarized the relationship between each lncRNA and clinicopathologic characteristics such as age, gender, smoking history, histological type, tumor size, and lymph node metastasis (Supplementary Table 2). We then calculated the pooled odds ratios (ORs) and 95% CIs according to the different clinicopathological features. We found that lncRNA expression showed no correlation with age [13–15, 17, 18, 20–30, 32–34, 36, 42, 44–46, 48–53], gender [13–18, 20–30, 32–36, 39, 42, 44–46, 48–53], smoking history [13, 17, 18, 20, 23–26, 28, 29, 32, 33, 35, 36, 42, 45, 46, 48, 49, 51, 53], or histological type [14–18, 20, 24, 25, 28, 29, 32–34, 36, 39, 42, 44–46, 48, 49, 52, 53], but that it showed a close relationship with tumor size [13–18, 20–29, 32–34, 36, 42, 44–46, 48–53] and lymph node metastasis [13–18, 20–29, 32–36, 42, 44–46, 49–53]; i.e., high expression of oncogenic lncRNAs was associated with poorer pathological parameters, while high expression of tumour-suppressor lncRNAs showed the opposite. The pooled ORs and 95% CIs of the relationship between lncRNA expression and clinicopathological characteristics were summarized in Table 1. The corresponding forest plots were shown in Supplementary Figure 1.

**Table 1:** Pooled odds ratios and 95% confidence intervals for clinical characteristics of lncRNAs in NSCLC

	Tumor promoter		Tumor suppressor	
	OR	95% Cl	OR	95% CI
Age				
cut off, 60	0.95	0.72–1.25	0.85	0.54–1.34
cut off, 65	0.93	0.69–1.25	0.85	0.55–1.32
Gender				
W/M	1.13	0.94–1.35	0.92	0.67–1.27
Smoking history				
Y/N	1.16	0.79–1.70	1.26	0.86–1.84
Histological type				
Ade/Squ	0.94	0.74–1.18	1.27	0.91–1.77
Tumor size				
cut off, 3 cm	2.74	1.66–4.52	0.33	0.14–0.75
cut off, 5 cm	2.04	1.09–3.79	0.28	0.18–0.45
Lymph node metastasis				
Y/N	3.3	2.42–4.49	0.37	0.26–0.52

W, women; M, men; Y, yes; N, no; Ade, adenocarcinoma; Squ, squamous cell carcinoma.

### LncRNA may have utility as a serum biomarker for NSCLC

**Figure 3:**
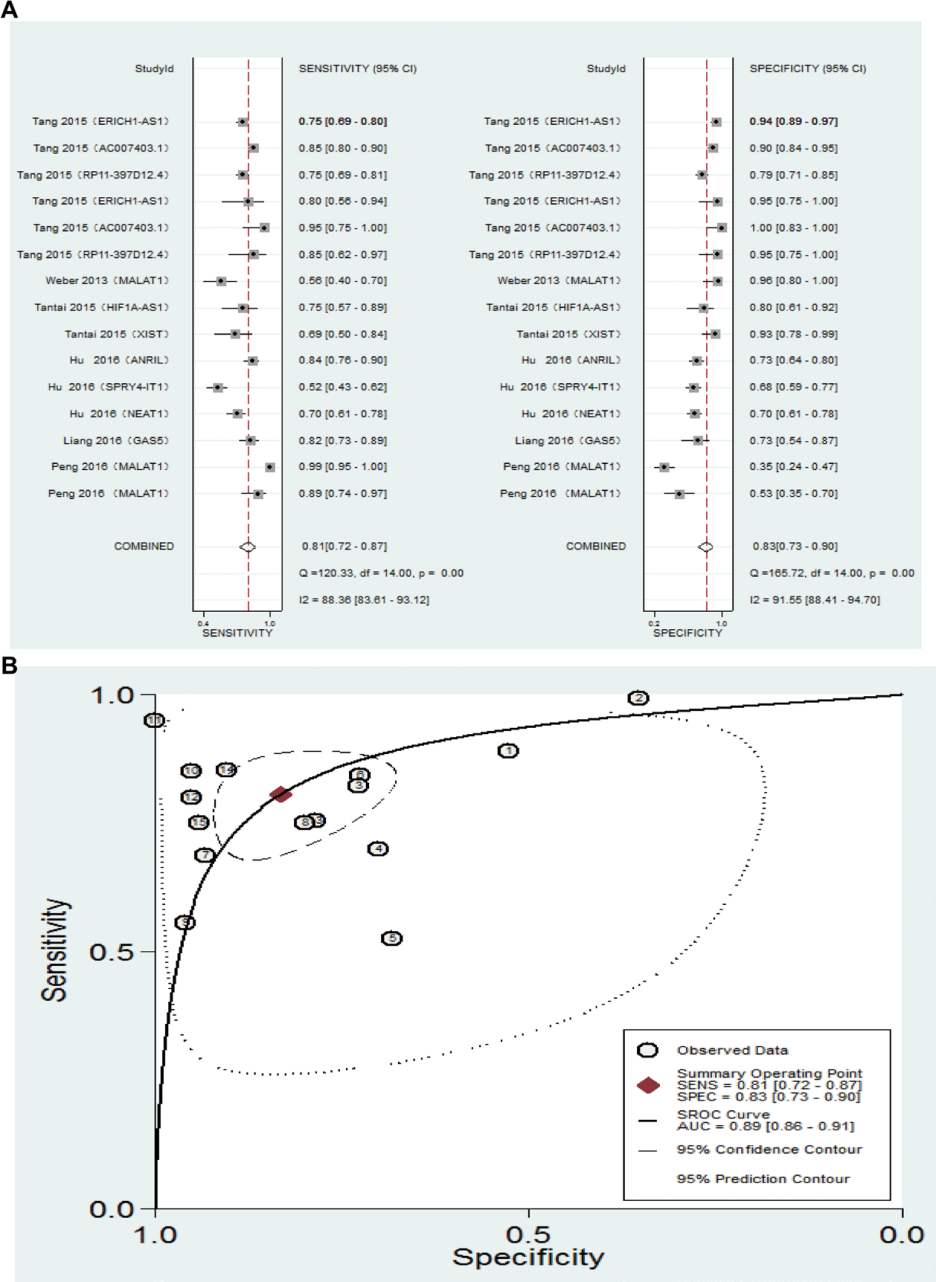
(**A**) Forest plot showing the sensitivity and specificity of serum lncRNAs for a diagnosis of NSCLC. (**B**) Summary Receiver operating characteristics curve for lncRNAs.

To investigate the diagnostic utility of lncRNA for NSCLC, we first identified studies (six studies [54–59]) reporting that ten lncRNAs may be useful as serum markers of NSCLC. We summarized the trial name, lncRNA type, data type, area under the curve (AUC), *p* values, 95% CIs, and sensitivity and specificity in Supplementary Table 3. Next, we estimated the pooled indices for these diagnostic reference values, while Spearman correlation coefficient ruled out the threshold effect (r = 0.104 , *p* = 0.713). The results were as follows: sensitivity, 0.81 [0.72–0.87]; specificity, 0.83 [0.73–0.90]; positive likelihood ratio, 4.8 [3.0–7.8]; negative likelihood ratio, 0.23 [0.16–0.34]; and overall diagnostic OR, 21 [11–39]. The forest plot was shown in Figure 3A. The summary receiver operator characteristic curve (SROC) AUC value was 0.89 (0.86–0.91), indicating that lncRNAs have a high diagnostic accuracy (Figure 3B).

### LncRNAs affect malignant progression of NSCLC by regulating signaling pathways via gene expression modulation

**Figure 4:**
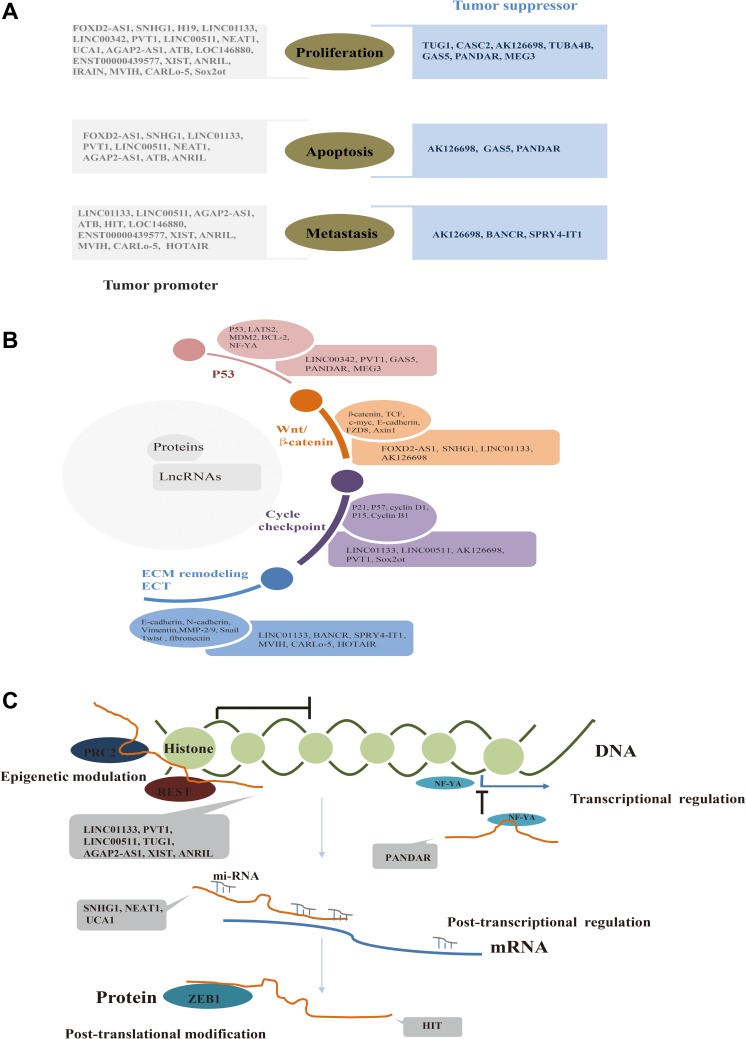
(**A**) LncRNAs function as tumor promoters or suppressors by regulating malignant characteristics. (**B**) LncRNAs regulate classical signaling pathways or biological processes critical for NSCLC by regulating critical proteins. (**C**) LncRNAs regulate almost every step of gene expression.

To further examine the link between lncRNAs and NSCLC, we examined their effect on the malignant phenotype. We found that lncRNAs regulated cell proliferation, apoptosis, and metastasis. Some lncRNAs affected all three of these cancer hallmarks, whereas some affected only one (Figure 4A). Furthermore, we found that lncRNAs regulated expression of key proteins within signal networks or biological processes (e.g., P53, Wnt/ β-catenin signaling, cell cycle checkpoints, epithelial mesenchymal transition (EMT), and extracellular matrix (ECM) remodeling), all of which regulate the malignant characteristics of cancer cells (Figure 4B). In addition, we examined how lncRNAs regulate gene expression; we found that lncRNAs function at epigenetic, transcriptional, post-transcriptional, and post-translational levels to modulate gene expression (Figure 4C). Taken together, the data suggested that lncRNAs functioned at every step of gene expression to mediate levels of key proteins. This in turn could impact molecular signaling pathways, leading to promotion or inhibition of malignant progression.

## DISCUSSION

Here, we systematically reviewed 37 eligible records to estimate the clinical utility of lncRNAs as diagnostic and prognostic indicators for NSCLC. We found that lncRNAs may be useful biomarkers for NSCLC. Further, we summarized the molecular mechanisms underlying involvement of lncRNAs in malignancy of NSCLC. Thus, lncRNAs are deserving of much attention (alongside proteins and DNA) in terms of their role in the pathogenesis of NSCLC.

Almost 70% NSCLC were diagnosed in an advanced state, which means limitation in medical intervention and poor prognosis, where efficient biomarker for predicting urgently needed [1, 5]. LncRNA is an emerging star in cancer research for its powerful function in modulating cellular processes and numerous studies have revealed the specific lncRNAs in NSCLC prognosis and diagnosis [7, 10, 11, 60, 61]. In our research, we systematically reviewed the function of lncRNAs in NSCLC. First, we found that lncRNAs had a close relationship with clinicopathological features of NSCLC, which consists of survival state, tumor size and lymph node metastasis. What we found proved the potency of lncRNAs in evaluating the clinical stage of the tumor and telling the prognosis of NSCLC, which may help us handle such malignancy in a novel perspective not the traditional invasive way. Further, we found that lncRNAs had high diagnostic efficiency in NSCLC, which may provide a convenient, economic and microinvasive approach to screen the NSCLC via this liquid biopsy.

Moreover, we deeply investigated the molecular underpinning of lncRNAs associated with NSCLC. As we know, NSCLC is a disease caused and promoted by defects in genes that activate or inactivate signal networks inside and outside the cells [62], where lncRNAs could regulate almost every step of gene expression, whose aberrance could trigger cellular chaos, facilitating malignancy indeed [8, 10]. In this manuscript, we found lncRNAs could regulate a series of critical tumor associated genes like P53, cyclinD1, β-catenin, E-cadherin and etc. involving in signaling networks like P53 pathway, cycle checkpointing group, Wnt/ β-catenin signal transduction cascades and EMT, ECM remodeling systems, which could modulate proliferation, survival and metastasis. Further, we focused on the gene expression modulation function of lncRNAs and we found lncRNAs involved in NSCLC could participate in epigenetic, transcriptional, post-transcriptional and post-translational modification via its interaction with chromatin, DNA, RNA, and protein.

However, limitations still exist,which could cause bias and heterogeneity. For example, insufficient samples, limited publication of negative results and language restrictions all could cause bias [63–65]; Next, due to inherent weakness in observational trial, group characters difference, trial plan difference and evaluation means difference all could cause higher heterogeneity [64, 66]; Last but not least, the complex function of lncRNAs, high heterogeneity of NSCLC, and unknown mechanisms in carcinogenesis all could affect our deducation [4, 61]. Where a more comprehensive and deeper research consisting of sufficient samples, multi-centers, higher quality studies with unified criteria, and etc. should be explored in the future work.

In conclusion, lncRNAs orchestrate gene expression at almost every step, resulting in activation or suppression of oncogenes and tumor suppressor genes, and ultimately affecting malignant progression via regulating signal networks. In NSCLC, such aberrant lncRNAs may have utility as novel diagnostic and prognostic indicators, which may point the way to new preventative and therapeutic options.

## MATERIALS AND METHODS

### Date sources and search strategy

PubMed, Web of Science, and Google Scholar were probed using the search term “ lncRNA OR long non-coding RNA OR lncRNAs OR long non-coding RNAs and NSCLC OR non-small cell lung cancer ”. The time window started at the time of database inception and extended to 1 April 2017. The language was limited to English.

### Inclusion and exclusion criteria

The inclusion criteria were as follows: (1) all patients diagnosed with NSCLC; (2) all studies used well-established methods of measuring and analyzing lncRNA (e.g., qPCR); (3) studies investigated the association between lncRNA and cancer prognosis with OS as a definitive outcome or other clinicopathological parameters like tumor size, lymph node metastasis and etc.. . Exclusion criteria were as follows: (1) reviews, case reports, or meeting presentations; (2) unqualified patients and grouping methods; (3) insufficient data to accurately calculate HRs, ORs and corresponding 95% CIs; (4) where duplicate publications or the same research was published in different formats, only the latest article was included.

Databases were examined by two independent investigators, and studies were selected based on title, abstract, and/or full text. Any disagreements were discussed and resolved by consensus.

### Date extraction and quality assessment

The date of each study was extracted, along with the name of the first author, the publication date, the number of samples, lncRNA examination methods and expression patterns, prognostic outcome (HR and 95% CI) and clinicopathological parameters. All extractions were performed by two independent investigators, and any disagreement was resolved by consensus. Furthermore, the quality of each study was assessed using the Newcastle-Ottawa Scale criteria or Quality Assessment for Diagnostic Accuracy Studies based on the type of clinical trial. This was again carried out by two independent investigators, and any disagreement was resolved by consensus. The assessment criteria included cohort selection, comparability, expose, and outcome.

### Statistical analysis

Data were analyzed using STATA software, version 12.0 (STATA Corporation, College Station, Texas, USA). HRs and corresponding 95% CIs were used to estimate the correlation between lncRNA expression and clinical prognosis of NSCLC. HRs, ORs, and sensitivity and specificity (and their respective 95% CIs) were collected from the article (if available) or calculated based on available information (calculations were carried out according to previously reported methods [67, 68]). Heterogeneity was examined using the *Q* test and the I^2^ test. Heterogeneity was considered statistically significant at a *p* value < 0.05 or an I^2^ value > 50% (when a random-effect model was applied). Otherwise, a fixed-effect model was used.

## SUPPLEMENTARY MATERIALS FIGURE AND TABLES







## Abbreviations

(NSCLC): Non-small cell lung cancer
(lncRNAs): long non-coding RNAs
(HRs): hazard ratios
(CIs): confidence intervals
(OS): overall survival
(ORs): odds ratios
(EMT): epithelial mesenchymal transition
(ECM): and extracellular matrix
(PRC2): Polycomb Repressive Complex 2
(REST): RE1 Silencing Transcription Factor
(NF-YA): Nuclear Transcription Factor Y Subunit Alpha
ZEB1: (Zinc Finger E-Box Binding Homeobox 1)
