# Supplementary material for: Long non-coding RNAs function as novel predictors and targets of non-small cell lung cancer: a systematic review and meta-analysis
Source: Oncotarget. 2018 Jan 4;9(13):11377–86. doi: 10.18632/oncotarget.23994 (PMC5834293; doi:10.18632/oncotarget.23994)
Supplement: Supplementary file 1 [file oncotarget-09-11377-s001.pdf]

Long non-coding RNAs function as novel predictors and targets of non-small cell lung cancer: a systematic review and meta-analysis

SUPPLEMENTARY MATERIALS

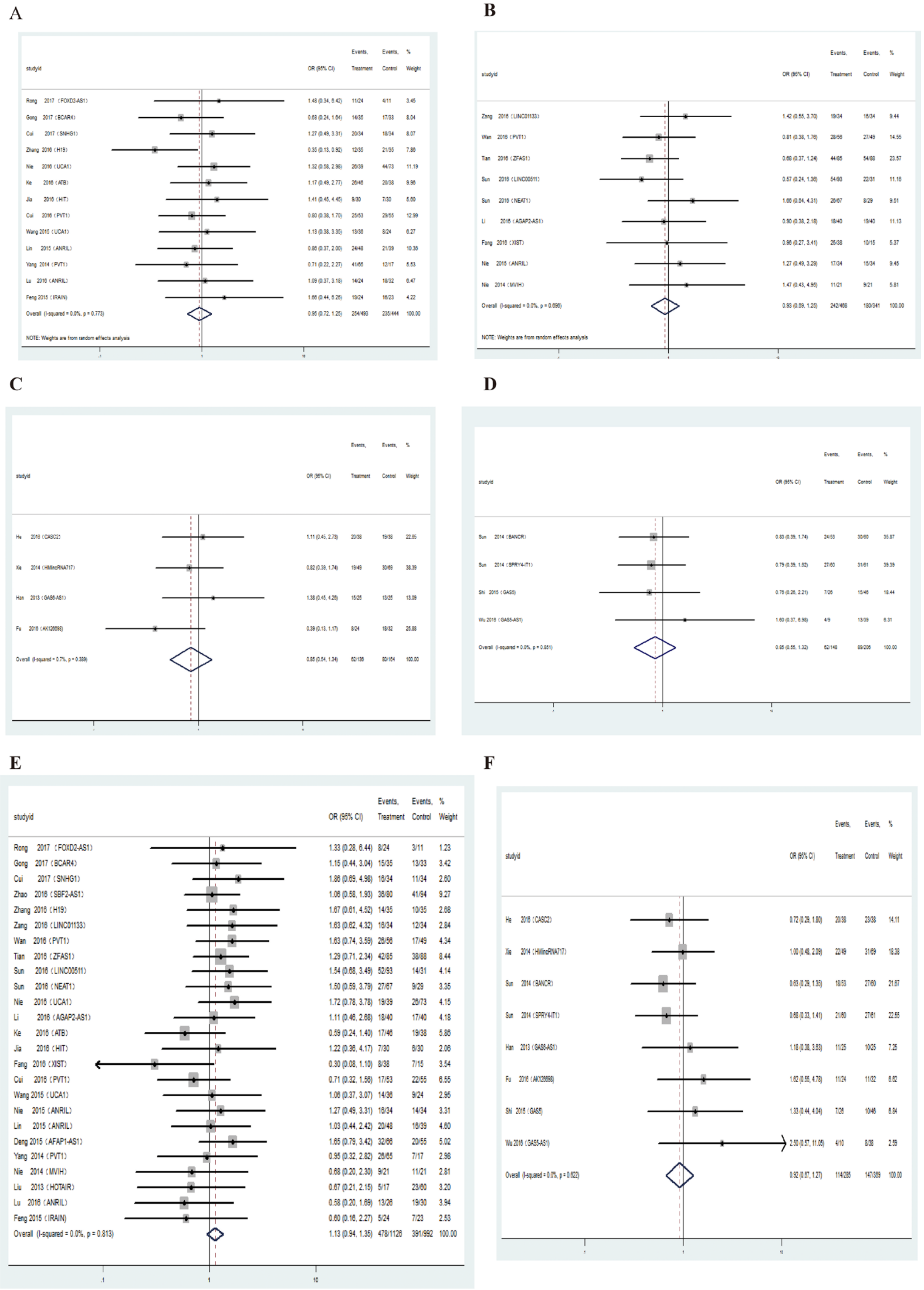

G

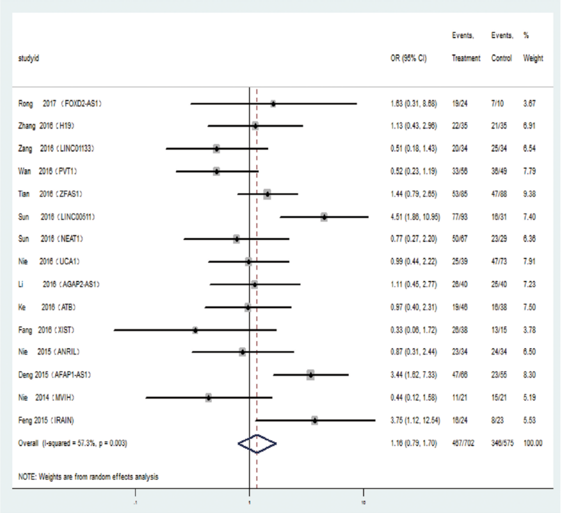
studyid
OR (95% CI)
Events, Treatment
Events, Control
% Weight
Rong 2017 (FOXD2-AS1) 1.63 (0.31, 8.68) 19/24 7/10 3.67
Zhang 2016 (H19) 1.13 (0.43, 2.96) 22/35 21/35 6.91
Zang 2016 (LINC01133) 0.51 (0.18, 1.43) 20/34 25/34 6.54
Wan 2016 (PVT1) 0.52 (0.23, 1.19) 33/56 36/49 7.79
Tian 2016 (ZFAS1) 1.44 (0.79, 2.65) 53/65 47/68 9.38
Sun 2016 (LINC00511) 4.51 (1.86, 10.96) 77/93 18/31 7.40
Sun 2016 (NEAT1) 0.77 (0.27, 2.20) 50/67 23/29 6.36
Nie 2016 (UCA1) 0.99 (0.44, 2.22) 25/39 47/73 7.91
Li 2016 (AGAP2-AS1) 1.11 (0.45, 2.77) 26/40 25/40 7.23
Ke 2016 (ATB) 0.97 (0.40, 2.31) 19/46 16/38 7.50
Fang 2016 (XIST) 0.33 (0.06, 1.72) 26/38 13/15 3.78
Nie 2015 (ANRIL) 0.87 (0.31, 2.44) 23/34 24/34 6.50
Deng 2015 (AFAP1-AS1) 3.44 (1.62, 7.33) 47/66 23/55 8.30
Nie 2014 (MVIH) 0.44 (0.12, 1.58) 11/21 15/21 5.19
Feng 2015 (IRAIN) 3.75 (1.12, 12.54) 16/24 8/23 5.53
Overall (I-squared = 57.3%, p = 0.003) 1.16 (0.79, 1.70) 457/702 346/575 100.00
NOTE: Weights are from random effects analysis
.1
1
10

H

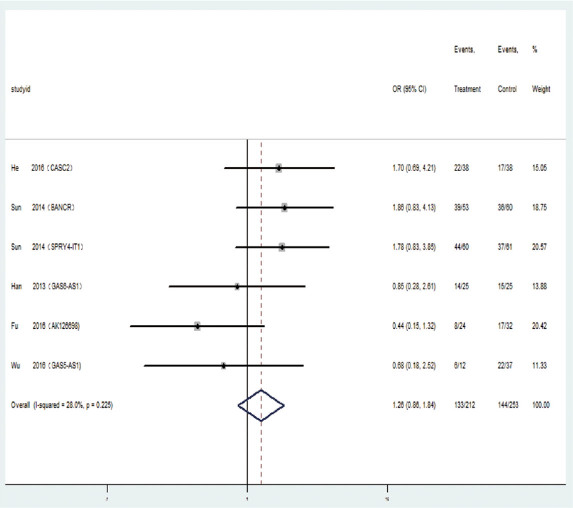
studyid
OR (95% CI)
Events, Treatment
Events, Control
% Weight
He 2016 (CASC2) 1.70 (0.69, 4.21) 22/38 17/38 15.05
Sun 2014 (BANCR) 1.86 (0.83, 4.13) 39/53 36/60 18.75
Sun 2014 (SPRY4-IT1) 1.78 (0.83, 3.85) 44/60 37/61 20.57
Han 2013 (GAS6-AS1) 0.85 (0.28, 2.61) 14/25 15/25 13.88
Fu 2016 (AK126698) 0.44 (0.15, 1.32) 8/24 17/32 20.42
Wu 2016 (GAS6-AS1) 0.68 (0.18, 2.52) 6/12 22/37 11.33
Overall (I-squared = 28.0%, p = 0.225) 1.26 (0.86, 1.84) 133/212 144/253 100.00
.1
1
10

I

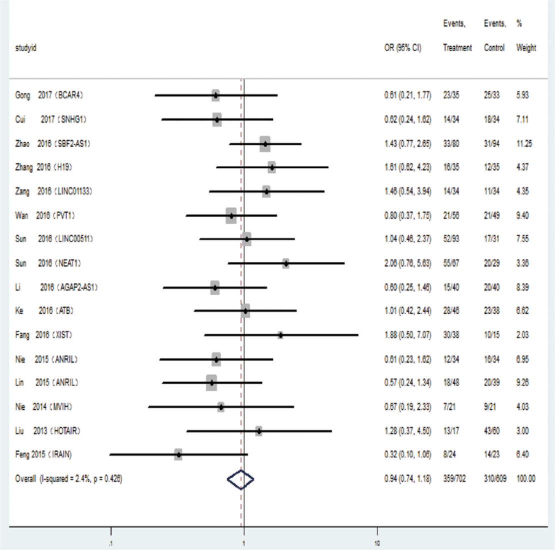
studyid
OR (95% CI)
Events, Treatment
Events, Control
% Weight
Gong 2017 (BCAR4) 0.61 (0.21, 1.77) 23/35 25/33 5.93
Cui 2017 (SNHG1) 0.62 (0.24, 1.62) 14/34 18/34 7.11
Zhao 2016 (SBF2-AS1) 1.43 (0.77, 2.65) 33/80 31/94 11.25
Zhang 2016 (H19) 1.61 (0.62, 4.23) 16/35 12/35 4.37
Zang 2016 (LINC01133) 1.46 (0.54, 3.94) 14/34 11/34 4.35
Wan 2016 (PVT1) 0.80 (0.37, 1.75) 21/56 21/49 9.40
Sun 2016 (LINC00511) 1.04 (0.46, 2.37) 52/93 17/31 7.55
Sun 2016 (NEAT1) 2.06 (0.76, 5.63) 55/67 20/29 3.38
Li 2016 (AGAP2-AS1) 0.60 (0.25, 1.46) 15/40 20/40 8.39
Ke 2016 (ATB) 1.01 (0.42, 2.44) 28/46 23/38 6.62
Fang 2016 (XIST) 1.88 (0.50, 7.07) 30/38 10/15 2.03
Nie 2015 (ANRIL) 0.61 (0.23, 1.62) 12/34 16/34 6.95
Lin 2015 (ANRIL) 0.57 (0.24, 1.34) 18/48 20/39 9.26
Nie 2014 (MVIH) 0.67 (0.19, 2.33) 7/21 9/21 4.03
Liu 2013 (HOTAIR) 1.28 (0.37, 4.50) 13/17 43/60 3.00
Feng 2015 (IRAIN) 0.32 (0.10, 1.06) 8/24 14/23 6.40
Overall (I-squared = 2.4%, p = 0.426) 0.94 (0.74, 1.18) 359/702 310/609 100.00
.1
1
10

J

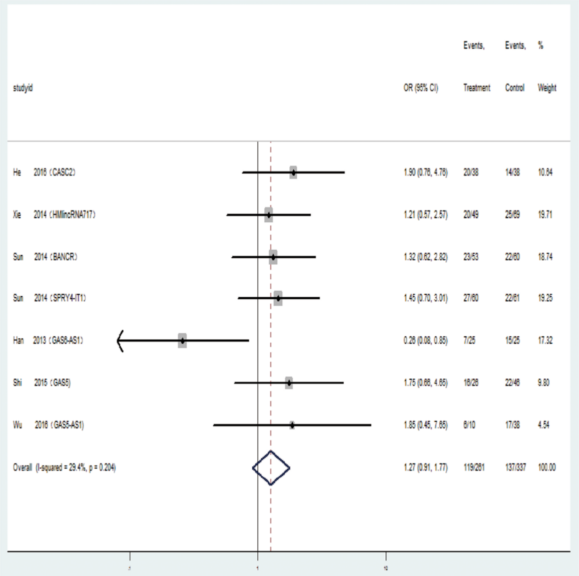
studyid
OR (95% CI)
Events, Treatment
Events, Control
% Weight
He 2016 (CASC2) 1.90 (0.76, 4.76) 20/38 14/38 10.94
Xie 2014 (HMlincRNA717) 1.21 (0.57, 2.57) 20/49 25/69 19.71
Sun 2014 (BANCR) 1.32 (0.62, 2.82) 23/53 22/60 18.74
Sun 2014 (SPRY4-IT1) 1.45 (0.70, 3.01) 27/60 22/61 19.25
Han 2013 (GAS6-AS1) 0.26 (0.08, 0.85) 7/25 15/25 17.32
Shi 2015 (GAS5) 1.75 (0.66, 4.65) 16/26 22/46 9.80
Wu 2016 (GAS6-AS1) 1.85 (0.45, 7.65) 6/10 17/38 4.54
Overall (I-squared = 29.4%, p = 0.204) 1.27 (0.91, 1.77) 119/261 137/337 100.00
.1
1
10

K

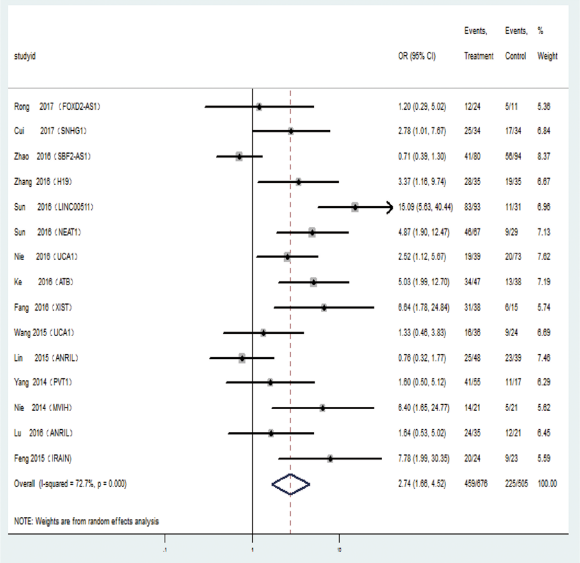
studyid
OR (95% CI)
Events, Treatment
Events, Control
% Weight
Rong 2017 (FOXD2-AS1) 1.20 (0.29, 5.02) 12/24 5/11 5.36
Cui 2017 (SNHG1) 2.78 (1.01, 7.67) 25/34 17/34 6.84
Zhao 2016 (SBF2-AS1) 0.71 (0.39, 1.30) 41/80 56/94 8.37
Zhang 2016 (H19) 3.37 (1.16, 9.74) 28/35 19/35 6.67
Sun 2016 (LINC00511) 15.09 (5.63, 40.44) 83/93 11/31 6.96
Sun 2016 (NEAT1) 4.87 (1.90, 12.47) 46/67 9/29 7.13
Nie 2016 (UCA1) 2.52 (1.12, 5.67) 19/39 20/73 7.62
Ke 2016 (ATB) 5.03 (1.99, 12.70) 34/47 13/38 7.19
Fang 2016 (XIST) 6.64 (1.78, 24.84) 31/38 6/15 5.74
Wang 2015 (UCA1) 1.33 (0.46, 3.83) 16/36 9/24 6.69
Lin 2015 (ANRIL) 0.76 (0.32, 1.77) 25/48 23/39 7.46
Yang 2014 (PVT1) 1.60 (0.50, 5.12) 41/55 11/17 6.29
Nie 2014 (MVIH) 6.40 (1.65, 24.77) 14/21 5/21 5.62
Lu 2016 (ANRIL) 1.64 (0.53, 5.02) 24/35 12/21 6.45
Feng 2015 (IRAIN) 7.78 (1.99, 30.35) 20/24 9/23 5.59
Overall (I-squared = 72.7%, p = 0.000) 2.74 (1.66, 4.52) 459/676 225/505 100.00
NOTE: Weights are from random effects analysis
.1
1
10

L

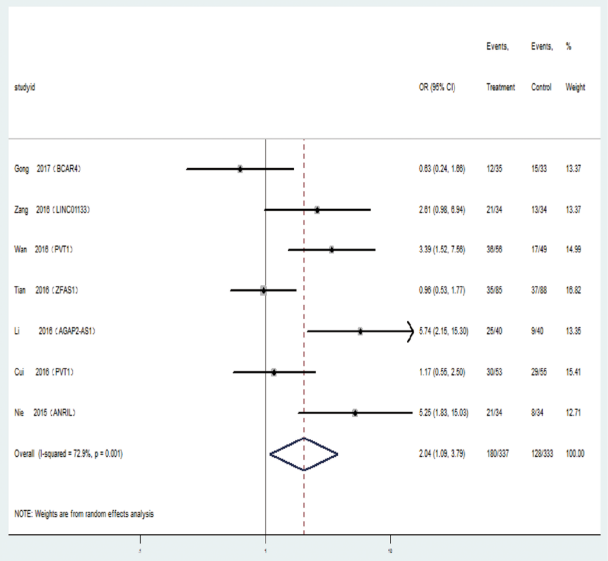
studyid
OR (95% CI)
Events, Treatment
Events, Control
% Weight
Gong 2017 (BCAR4) 0.63 (0.24, 1.66) 12/35 15/33 13.37
Zang 2016 (LINC01133) 2.61 (0.98, 6.94) 21/34 13/34 13.37
Wan 2016 (PVT1) 3.39 (1.52, 7.56) 38/56 17/49 14.99
Tian 2016 (ZFAS1) 0.96 (0.53, 1.77) 35/65 37/68 16.82
Li 2016 (AGAP2-AS1) 5.74 (2.15, 15.30) 25/40 9/40 13.36
Cui 2016 (PVT1) 1.17 (0.55, 2.50) 30/53 29/55 15.41
Nie 2015 (ANRIL) 5.25 (1.83, 15.03) 21/34 8/34 12.71
Overall (I-squared = 72.9%, p = 0.001) 2.04 (1.09, 3.79) 180/337 128/333 100.00
NOTE: Weights are from random effects analysis
.1
1
10

M

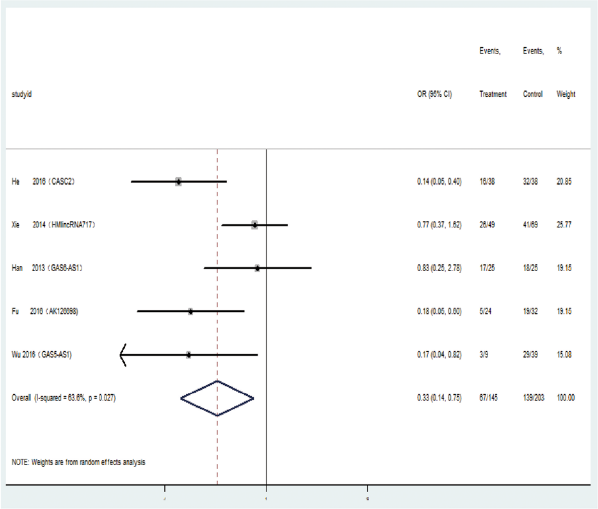

N

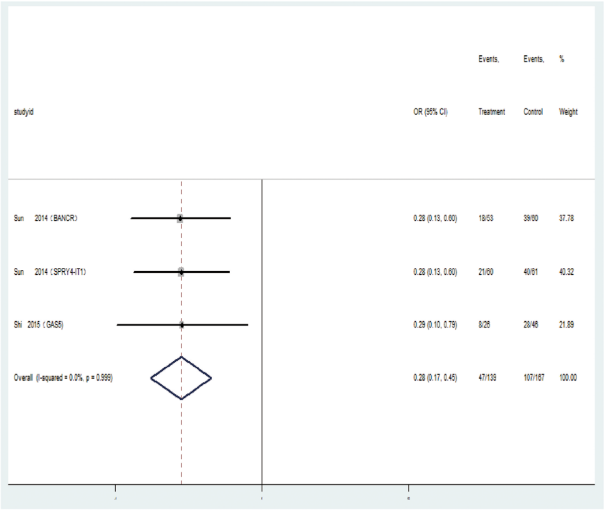

O

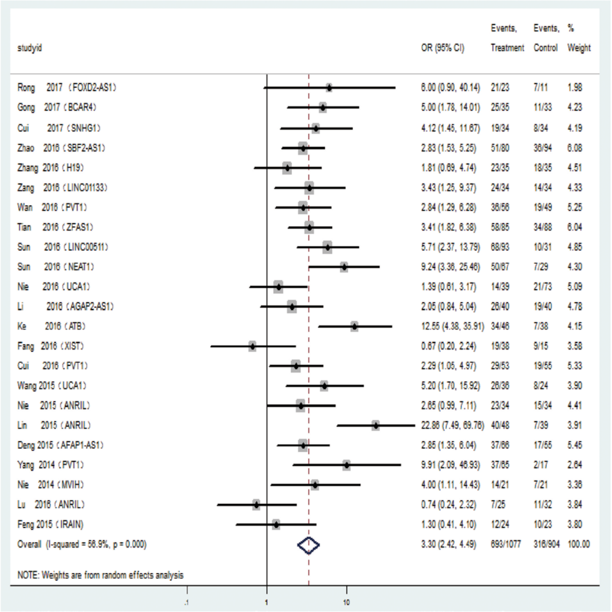

P

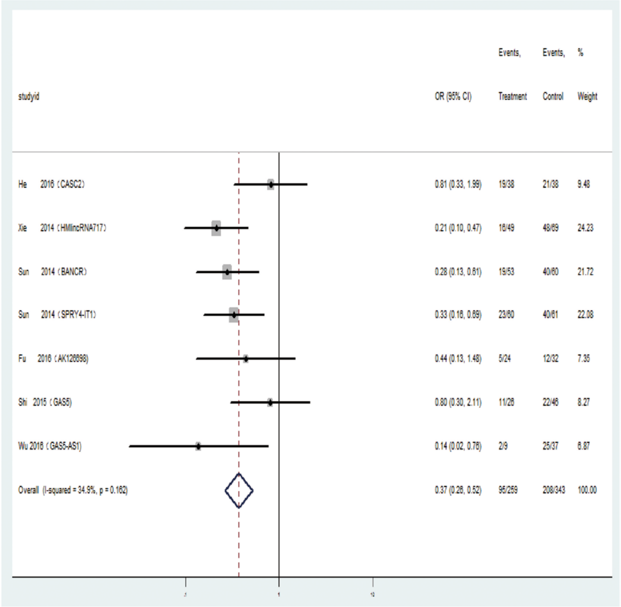

Supplementary Figure 1: Forest plot showing the odds ratios and 95% confidence intervals (age (**A**) Cut value is 60 years old for oncogenic lncRNAs, (**B**) Cut value is 65 years old for oncogenic lncRNAs, (**C**) Cut value is 60 years old for tumour suppressor lncRNAs, (**D**) Cut value is 65 years old for tumour suppressor lncRNAs; gender (**E**) oncogenic lncRNAs, (**F**) tumour suppressor lncRNAs); smoking history (**G**) oncogenic lncRNAs, (**H**) tumour suppressor lncRNAs), histological type (**I**) oncogenic lncRNAs; (**J**) tumour suppressor lncRNAs), tumor size (**K**) Cut value is 3 cm for oncogenic lncRNAs, (**L**) Cut value is 5 cm for oncogenic lncRNAs, (**M**) Cut value is 3 cm for tumour suppressor lncRNAs, (**N**) Cut value is 5 cm for tumour suppressor lncRNAs; and lymph node metastasis (**O**) oncogenic lncRNAs, (**P**) tumour suppressor lncRNAs.)

Supplementary Table 1: Relationship between OS and lncRNAs in NSCLC. See Supplementary_Table_1

Supplementary Table 2: Relationship between lncRNAs and the clinical pathological parameters of NSCLC. See Supplementary_Table_2

Supplementary Table 3: Diagnostic parameters about relationship between lncRNAs and NSCLC

Trial name	LncRNA type	Data type	AUC	P	95% CI	Sensitivity	Specificity	NSCLC	Normal
Peng 2016	MALAT1	blood sample	0.708	0.002	0.586–0.830	0.889	0.528	36	36
Peng 2016	MALAT1	blood sample	0.672	< 0.001	0.587–0.757	0.992	0.352	120	71
Liang 2016	GAS5	blood sample	0.832	< 0.0001	0.754–0.893	0.822	0.727	90	33
Hu 2016	NEAT1	blood sample	0.693	< 0.001	0.619–0.768	0.696236	0.697599	120	120
Hu 2016	SPRY4-IT1	blood sample	0.603	0.012	0.525–0.682	0.523416	0.686007	120	120
Hu 2016	ANRIL	blood sample	0.798	< 0.001	0.735–0.862	0.843948	0.723137	120	120
Tantai 2015	XIST	blood sample	0.834	< 0.001	0.726–0.935	0.685484	0.9274496	32	30
Tantai 2015	HIF1A-AS1	blood sample	0.876	< 0.001	0.793–0.965	0.762646	0.814318	32	30
Weber 2013	MALAT1	blood sample	0.79	NA	0.68–0.89	0.56	0.96	45	25
Tang 2015	RP11–397D12.4	blood sample	0.9	< 0.001	0.791–1.009	0.854348	0.9478211	20	20
Tang 2015	AC007403.1	blood sample	0.974	< 0.001	0.916–1.032	0.956413	1	20	20
Tang 2015	ERICH1-AS1	blood sample	0.875	< 0.001	0.755–0.955	0.800317	0.945994	20	20
Tang 2015	RP11–397D12.4	blood sample	0.77	0.004	0.615–0.924	0.752351	0.782566	232	135
Tang 2015	AC007403.1	blood sample	0.868	< 0.001	0.745–0.992	0.851887	0.9001557	232	135
Tang 2015	ERICH1-AS1	blood sample	0.849	< 0.001	0.718–0.979	0.750971	0.9421792	232	135

NA (not available).
